# Identification of decision-making criteria for device selection by cochlear implant patients – a pilot study

**DOI:** 10.1007/s00405-025-09867-y

**Published:** 2026-02-01

**Authors:** Stefanie Bruschke, Timo Stöver, Silke Helbig, Uwe Baumann

**Affiliations:** ENT Department, Goethe University Frankfurt, University Hospital, Theodor-Stern-Kai 7, 60590 Frankfurt a. M, Germany

**Keywords:** Cochlear implant, Patient counselling, Decision criteria, Device selection, Pilot study

## Abstract

**Purpose:**

Prior to a cochlear implant (CI) treatment, patients undergo an extensive consultation process. In Germany, patients themselves usually can select a device from one of the offered CI manufacturers. Patients therefore receive a large amount of information, to support the challenging decision-making process. The aim of the study was to evaluate the parameters that are most important to patients during the consultation process for choosing a CI device.

**Methods:**

In total, 41 patients were included in our study. Questionnaires were used to assess the subjective importance of various device related parameters, such as features of the implant, processor, hearing programs and accessories for the counseling process for selection of the CI device. A Likert scale was used to assess the importance of the respective parameters (“very important”, “important”, “rather unimportant” and “unimportant”). The assessment was done at three time points: preoperatively (day of device selection), approx. three weeks postoperatively (after completion of the initial fitting) and after six months of experience with CI use.

**Results:**

The “implant reliability” and the “processor wearing comfort” were rated as very important at all observation points. The availability of a “noise reduction” algorithm was also rated as important. The features of the implant, such as the “shape of the electrode” and “length of the electrode array”, as well as features of the remote control, such as the “availability of a display”, were rated as less important content during the counseling for device selection. The rating for some parameters, e.g. subjective importance, has even changed over time. The availability of “directional microphone technique” and the ”availability of processor buttons” was rated more important postoperatively with CI usage experience than preoperatively.

**Conclusion:**

Based on the study results, important and unimportant parameters of the CI system during the counseling process were identified. It should be taken into account that several parameters important to the patient may change over time with increasing practical CI experience. Our data may be useful in optimizing the CI counseling process. Although CI counseling must follow defined quality standards, it remains an individualized process that must take into account the specific needs of the patient.

## Introduction

The cochlear implant (CI) is the established standard of care for patients with severe to profound hearing loss and completely deaf patients, if a conventional hearing aid does not provide sufficient speech perception [1]. Most CI patients are able to improve speech perception significantly and additionally report an enhanced quality of life in everyday situations [2–4].

Prior to the CI surgery, patients undergo an evaluation process to assess their candidacy. According to the German CI guidelines (AWMF CI guideline [5], CI Whitebook [6]) this includes a clinical ENT examination as well as various audiological tests. In addition, clinical diagnostics of vertigo and imaging diagnostics using CT (computed tomography) and MRI (magnetic response imaging) are performed. Also, as part of the preliminary counseling process, a detailed medical and technical-audiological consultation takes place [6]. The medical consultation is focused on the surgical procedure and associated potential risks, while the audiological-technical consultation aims to explain the basic functional aspects of the CI. Additionally, alternative treatment options and the postoperative rehabilitation process are presented. If there are no audiological or medical reasons to recommend a specific device (e.g. difficult surgical access to the cochlear), our patients usually decide themselves which CI device to choose. All CI systems are covered equally by the coast bearers. In the authors’ clinic, surgical or audiological recommendations for a specific electrode type are provided in selected cases. For certain conditions, such as otosclerosis or cochlear ossification following meningitis, more robust electrodes that can be inserted with the aid of a stylet (e.g. CI512/CI612, Cochlear) are recommended. These electrode types are also preferred from a surgical perspective in cases of cochlear malformations. In patients with sufficient low-frequency residual hearing preoperatively, which can benefit from an electric-acoustic stimulation (EAS), shorter electrode arrays are advised in order to establish the preservation of low-frequency hearing. In the absence of surgical or audiological recommendations, patients at the authors’ clinic are free to choose which CI system they wish to use. As a consequence, the consultation in addition provides detailed specific information on all CI devices, regarding technical specifications (e.g. “implant reliability”, “processor size”, “noise reduction”, “compatibility with smartphone”). The consultation thus also aims to provide patients with additional information to support their device selection process. To enable this decision-making process, patients receive manufacturer-specific information material for further counseling. In addition, an overview of the manufacturers is also provided to CI patients during their first consultation. This overview was created by the authors’ clinic in collaboration with the respective CI manufacturers and is regularly revised. It compares the technical parameters of the CI system for the implant (e.g. size and weight, electrode types) and the processor (e.g. size and weight, compatible devices, available accessories). This overview is then discussed with the patients during the appointment to select the CI system. The preoperative consultation is carried out according to a standardized internal protocol, covering the above-mentioned aspects and ensuring equal information to all patients being counseled prior to CI therapy.

The CI therapy in Germany is highly standardized due to a national CI clinical practice guideline [5], a CI Whitebook [6] and even a recently established certification process for quality control [7]. As part of the quality control of the counseling process, CI candidates have to be offered CI devices of at least three different manufacturers. In our department four different CI manufacturers were implanted during the study period (2017–2020): Advanced Bionics, Cochlear, MED-EL and Oticon Medical. Due to the continuous further development of the implant / processor technology, connectivity as well as external accessories (e.g. additional microphones), the consultation and device selection process has become increasingly complex. Patients have to choose a device based on a huge load of information. Thus, the decision process for patients often is challenging. In many cases it is uncertain which information content is of highest relevance to a patient during the consultation process. The potential benefit of some features, such as an algorithm for noise reduction, may not be valued by all patients in advance of the CI surgery, but can only be assessed later with sufficient CI experience. Thus, it is also conceivable that personal preferences likely change with CI usage experience.

The aim of the present study is to identify criteria that are of paramount importance to patients during the preoperative counseling and decision-making process. In addition, we assumed that preoperatively important counseling items may change postoperatively over time with CI experience. These results will be helpful in order to optimize the counseling process prior to the CI treatment to benefit the patients need.

## Materials and methods

The prospective study included 41 patients (20 male, 21 female) with a mean age of 61.4 years (min. 25 years / max. 88 years). Inclusion criteria for study participation was an age of at least 18 years, as well as a scheduled CI treatment. The exclusion criterion was a (bilateral) CI surgery on the second side, as these patients were already experienced with a certain CI device. The patients were treated with a CI in the period from 2017 to 2020. In total, 25 patients have chosen a BTE-processor which is worn behind the ear and 16 patients decided for a single-unit processor that is placed above the implant. The majority of patients (*n* = 28) used a CI-system from the manufacturer *Cochlear* with a *CI512* electrode array *(n* = 19*)*. Table 1 provides an overview of the CI systems used by the patients. Questionnaires were developed to evaluate important information for the counseling process prior to the CI device selection. These were handed out at three time points: preoperatively (day of device selection), approx. three weeks postoperatively (after completion of the initial fitting) and at the regular follow-up visit after six months following CI surgery (6 M). Using the questionnaires, the patients were asked to use a Likert scale [8] to rate the importance of various parameters during the counseling process for CI device selection. A choice could be made between “very important”, “important”, “rather unimportant” and “unimportant”. The questionnaire was divided into different sections and included implant characteristics such as “implant reliability”, “shape of the electrode” and “implant size”, as well as processor characteristics such as “processor wearing comfort” and “processor size”. In addition, features of the hearing programs, e.g. “directional microphone technique”, “noise reduction” and characteristics of the remote control, e.g. “availability of a display on the remote control”, as well as accessory characteristics, such as “compatibility with smartphone” or “swim protector”, were recorded. A summary of all the parameters evaluated with the questionnaires is shown in Table 2.

**Table 1:** CI systems used by the study participants, BTE – behind the ear

Manufacturer	Cochlear (*n* = 28)	MED-EL (*n* = 11)	Advanced Bionics (*n* = 2)	
Processor type				
BTE system	CP1000 (*n* = 17)	Sonnet (*n* = 6)	Naida Q90 (*n* = 2)	
Single-unit processor	Kanso (*n* = 11)	Rondo2 (*n* = 5)		
Implant type	CI512 (*n* = 19) CI522 (*n* = 4) CI532 (*n* = 2) CI612 (*n* = 3)	Synchrony 2 FlexSoft (*N* = 8) Synchrony Flex28 (*n* = 8) Synchrony Flex26 (*n* = 1) Synchrony Flex24 (*n* = 1*)*	HiRes90K Ultra Midscala (*n* = 1) HiRes90K 3D Midscala (*n* = 1)	

**Table 2:** Listing of all parameters in the questionnaire in order to evaluate the patients’ decision-making criteria

Implant characteristics	- implant size - length of the electrode array - number of electrodes - MRI properties - implant reliability - shape of the electrode - number of electrodes
Processor characteristics	- processor size - shape of processor - processor color selection - size of transmitting coil - processor wearing comfort - placement of the processor - microphone position - simplicity of processor handling - availability of processor buttons
Features of the hearing programs	- number of program slots - directional microphone technique - noise reduction - program for automatic detection of the listening situation
Remote control characteristics	- size of the remote control - remote control clarity - availability of a display on the remote control - possibility to adjust some parameters via remote control
Accessory characteristics	- type of wireless accessory - compatibility with smartphone - swim protector

The provided data were ranked based on the results of the questionnaires on the various parameters. In each case, a ranking list was created with the five parameters that the patients considered most important and least important during the CI counseling process for CI device selection. The ranking lists were created for each of the three time points (preoperatively, three weeks postoperatively, six months postoperatively). This enables to evaluate changes in the preferences over time with CI experience.

### Statistical evaluation

Only complete data sets were used for data analysis. The response categories of the Likert scale were converted into points. The statement “very important” corresponds to 4 points, “important” 3 points, “rather unimportant” 2 points and “unimportant” 1 point. The results from all patients regarding the subjective assessment of the category importance were summed up for the respective items and the corresponding ranking list with the top five ranks with the highest point values were created. In addition the bottom five ranks with the lowest point values were identified. Also the three ranks that presented the greatest change over time were extracted.

## Results

The results of the subjective assessment of the importance of various CI parameters were presented in form of ranking lists.

### Parameters rated as most important

The parameters rated most important by the patients are listed in Table 3. “Implant reliability” was rated most important at all time points, both preoperatively and postoperatively after initial fitting phase and at 6-month visit (rank 1). The “processor wearing comfort” was also rated as important at all time points (rank 2). This was followed by the opportunity of using a “noise reduction” algorithm (rank 3). “Simplicity of processor handling” is another parameter that was almost equally important rated by patients, both preoperatively (rank 4) and postoperatively (IF and 6 M - rank 3).

**Table 3:** Parameters rated by patients as most important for CI device selection

Rank	*P*	After selection of the implant system	*P*	After first fitting	*P*	at 6-month visit
1	155	implant reliability	152	implant reliability	158	implant reliability
2	154	processor wearing comfort	150	processor wearing comfort	150	processor wearing comfort
3	146	noise reduction	147	simplicity of processor handling; noise reduction	145	simplicity of processor handling; noise reduction
4	137	simplicity of processor handling	131	processor size; program for automatic detection of the listening situation	127	type of wireless accessory
5	127	implant size	125	placement of the processor; directional microphone technique	126	processor size; microphone position; program for automatic detection of the listening situation

Listing by ranks; *P *points, time of observation: preoperatively after selection of the CI device; postoperatively after completed initial fitting, at 6-month follow-up visit

While preoperatively the “implant size” was rated as important (rank 5), postoperatively the “processor size” was rated as important (IF – rank 4, 6 M – rank 5). Furthermore the availability of a “program for automatic detection of the listening situation” was rated important postoperatively (IF – rank 4, 6 M – rank 5).

## Parameters rated to be least important

The parameters of least importance to the patient are listed in Table 4. Both preoperatively and postoperatively the “length of the electrode array” (preoperative – rank 1, postoperative IF – rank 4, 6 M – rank 3) and the “shape of the electrode” (preoperative – rank 3, postoperative IF – rank 1, 6 M – rank 2) were rated as unimportant. Furthermore, features of the remote control were assessed as being of little importance at all observation times. In preoperative data collection, the “availability of processor buttons” was rated as unimportant by the patients (rank 2). In addition postoperatively the availability of a “swim protector” was rated as unimportant (IF – rank 3, 6 M – rank 1).

**Table 4:** Parameters assessed by the patients as most unimportant for CI device selection

Rank	*P*	After selection of the implant system	*P*	After first fitting	*P*	at 6-month visit
1	70	length of the electrode array	84	shape of the electrode	79	swim protector
2	72	availability of processor buttons	95	number of electrodes	80	shape of the electrode
3	74	shape of the electrode	98	swim protector	107	availability of a display on the remote control; size of the remote control; length of the electrode array
4	76	availability of a display on the remote control	99	length of the electrode array	109	compatibility with smartphone; availability of processor buttons
5	86	possibility to control some parameters via remote control	100	size of the remote control	110	processor color selection

Listing by ranks; *P* points, time of observation: preoperatively after CI device selection; postoperatively after completed initial fitting, at 6-month follow-up visit

## Changes in the subjective parameter assessment over time

The results further show that the importance of some parameters has changed over time (see Table 5). There were some parameters that were considered more important postoperatively than preoperatively. For example, the “possibility to adjust some parameters via remote control” (e.g. loudness level) was rated more important postoperatively with six months of CI experience than preoperatively. The “availability of processor buttons” and the ability to use “directional microphone technique” was also rated more important postoperatively with gained CI experience.

**Table 5:** Change in subjective importance of parameters over time, in brackets: change in ranks

Rank	Increase in importance	Decrease in importance
from system selection to first fitting phase		
1	directional microphone technique (12◊5); shape of processor (13◊6);	
2	remote control clarity (13◊8); availability of processor buttons (21◊16)	swim protector (15◊20); number of electrodes (16◊21)
3	possibility to control some parameters via remote control (18◊14)	Implant size (5◊9);
from system selection to 6-month visit		
1	possibility to control some parameters via remote control (18◊7)	
2	availability of processor buttons (21◊14) length of the electrode array (22◊15);	
3	microphone position (11◊5); directional microphone technique (12◊6)	
from first fitting phase to 6-month visit		
1	number of electrodes (21◊12)	
2	microphone position (12◊5); possibility to control some parameters via remote control (14◊7)	compatibility with smartphone (7◊14)
3	type of wireless accessory (10◊4)	

After 6 months of CI use, it was further observed that the “microphone position” as well as the ”type of wireless accessory” (external microphone, TV streamer, etc.) were subjectively more important to the experienced patients than those with little or no CI usage. However it was also possible to identify parameters that were rated less important by patients postoperatively with CI experience than preoperatively. These included the availability of a “swim protector” as well as implant characteristics such as the “number of electrodes” and the “implant size”.

## Discussion

In order to support patients to decide for a specific CI device, extensive consultations are carried out prior to a CI treatment. Due to the large amount of information, it is often difficult for patients to focus on the most important aspects. With the help of our study, parameters could be identified that were more or less important to patients during the CI counseling process.

### Parameters rated as most important

The “implant reliability” is particularly important to patients. Device failure would mean reimplantation with surgery followed by basic and follow-up therapy and rehabilitation to restore hearing performance [6]. In our study, data showed that the reliability of the cochlear implant stimulator was rated most important by the patients at all observation points. This component of the CI system remains in the patient’s body. Although at present, the survival rate of the CI stimulator devices after 10 years is about 98 %, regardless of the manufacturer and up to 95 % after 15 years [9] and the manufacturers provide a 10-year warranty [10], this item is clearly of great importance pre- and postoperatively.

Other parameters that were rated also very important by the patients at all observation times are the “processor wearing comfort” and the “simplicity of processor handling”. During the consultation process, the patient was informed that regular and consistent wearing of the processor is necessary in order to achieve adequate hearing benefit [11]. Thus, patients are aware that they will wear the processor permanently. Comfortable wearing and easy handling are therefore of great priority.

The availability of “noise reduction” was also rated important by the patients. Speech perception in noisy situations is a major challenge for many hearing-impaired people. The use of noise reduction algorithms improves speech perception in noisy environments [12]. Patients with hearing aid experience are already familiar with noise reduction mechanisms and their benefits for speech perception in more challenging listening situations.

Preoperatively, the “implant size” was rated as important by the patients. During the counseling process, patients are shown all CI stimulators in real size. Many patients prefer to have the smallest possible implant in their head.

Postoperatively, the “implant size” is no longer of great importance to the patients. Instead the “processor size” was rated more important with some CI experience. The processor is used daily and worn behind the ear (BTE) or over the implant (single unit processor). This may possibly be associated with restriction in everyday life, e.g. wearing glasses or helmet. The “processor size” is therefore an important aspect for patients postoperatively.

### Parameters rated to be least important

Some features of the implant were rated as unimportant by patients at all observation times. This included the “length of the electrode array” and the “shape of the electrode” (straight or modiolar preformed). These technical parameters appear to be very abstract for some patients. It may be assumed that the benefit of these parameters was not understandable for all patients and therefore was regarded as less important.

The features of the remote control were also rated as less important by the patients. The latest processor versions can also be connected to a smartphone app so that volume setting and programs can be adjusted, or audio streaming can be activated. The remote control is often left at home and the smartphone app is used to adjust the sound processor. The use of a remote control therefore usually is not very important for most patients in everyday life.

### Changes in the subjective parameter assessment over time

The assessment of the importance of some parameters for the counseling process has changed over time. Some parameters were rated as more important postoperatively than preoperatively. The use of accessories becomes more interesting for patients with some habituation to CI hearing and a corresponding improvement in speech recognition. Furthermore, once a certain stability of hearing has been achieved after 6 months [13], some patients seem to consider the possibility of adjusting hearing parameters themselves as important. Especially for patients with limited or no hearing aid experience, some parameters may be too technical or abstract to assess their importance prior to CI treatment. For example, the use of a “directional microphone technique” is rated more important postoperatively with CI experience than preoperatively. The benefit of this technology was experienced through the active use of the CI system.

Some parameters were evaluated more important preoperatively than postoperatively. The availability of a “swim protector” is not as important in the CI patient’s later daily life as it was during the preoperative consultations. When asked, the clear majority of patients stated that they do not use the swim protector in everyday life. It is usually more relevant for children or adult patients who are deaf on both ears and depend on hearing in water.

Implant characteristics, such as the “number of electrodes” and the “implant size”, were also less important postoperatively than preoperatively. After the successful implantation, such parameters no longer seem to be important for patients.

The results show that the importance as well as the unimportance of some parameters could only be judged with some experience in CI usage.

### Consequences for the counseling process

The consultation prior to a CI treatment is very extensive to patients, requiring a high level of attention. The listening effort during counseling is very high and a lot of information has to be processed. It can be assumed that at some point the patient’s attention may decrease. Much of the further information is hardly noticed by the patient. Optimizing the counseling sessions regarding patient-relevant information therefore may be useful.

Based on the study results, the consultation process prior to a CI treatment should be optimized. On the one hand, a patient has to get all decision-making relevant information, on the other hand, loss of attention and “overload” has to be avoided, as it may rather lead to distraction than additional value to the patient.

Our study was able to identify the most important aspects of the CI counseling process. For example, the “implant reliability” obviously is of particular importance to most patients. The benefits of those important parameters for patients should be explained in technical consultations, with emphasis to the persistent importance of these items.

Our study results also allowed to identify items being rated of less importance during the counseling process. For instance, this included the features of the implant. During consultations, patients are presented with examples of CI stimulators, but the advantage of a longer electrode, for instance, may be difficult for patients to appreciate. The study cannot determine whether the item in question was perceived as less valuable by patients due to its perceived lack of importance or if the patients did not fully comprehend the item’s significance. In future consultations, characteristics that were rated by patients as less important during the CI counseling process may be given less consideration than characteristics that were assessed as important by patients. This enables the amount of information conveyed in medical consultations to be focused.

An important outcome of this study is, that the relevance of some parameters changes over time. This should be considered in the consultation process and explained to the patient. It is of particular importance to direct attention to those parameters that were of greater significance to patients in the postoperative period than they could have foreseen in the preoperative period. It is of specific benefit to emphasize the advantages of these parameters. For example, the patient should be aware that the use of directional microphone technique can noticeably improve speech perception in noisy situations [14]. Parameters that appear to be of significance prior to surgery but are subsequently deemed irrelevant postoperatively may require less time during consultations. For instance, the availability of a swim protector is only briefly mentioned in the consultations. Since this is not an important aspect for the majority of patients, it is only discussed in individual cases.

The study data also provides the opportunity to reconsider the consultation material and the information provided to patients during CI counseling. The information should consider the essential interests of the patients, also with regard to a changing focus of the patients over time. It is not uncommon for patients to have a different assessment of the parameters than the specialist staff at clinical institutions. This discrepancy warrants further investigation and, if necessary, incorporation into the consultation and information process.

### Limitation of the study

Some CI device parameters are very technical, e.g. “length of the electrode array”. It is probable that these concepts were not fully identified and understood by all patients, thus preventing a comprehensive evaluation of these parameters. Furthermore, it was not determined how patients obtained information about different CI manufacturers, including internet sources, self-help groups, and information events, in addition to the regular consultation appointments in the clinic. These may had an influence on the assessment of important parameters for CI counseling. All patients who received a CI treatment at our department within the inclusion criteria were included in the study. The patient group then comprised 41 patients for whom complete data sets were available at all observation points. It was not considered whether the data was representative in terms of gender and age distribution. A possible bias in the selection of patients cannot therefore be ruled out. Although the number of patients included in the study is not insignificant, it cannot be definitively concluded that all relevant data has been obtained with the current sample size. Moreover, it is important to acknowledge that the type and content of CI counseling administered within our department can impact the outcomes, despite the use of established standards. This is yet an additional reason why the data might be different in different patient collective. It is also important to note that this study did not determine which parameters were decisive for the selection of the respective CI systems. Instead, it sought to identify which information was considered important in the context of CI counseling. In this study, patients who received a CI therapy were evaluated. Further studies should aim to gather information on why patients decided against CI therapy. Moreover, in a future follow-up study, the patients’ speech recognition could be evaluated after six months of CI use to investigate a possible correlation with the current study results. Nonetheless, the data represents an essential preliminary step; however, a larger number of patients could be included in future studies, potentially conducted in a multicenter setting.

## Conclusion

The study findings facilitate an enhanced counseling process through a reduction in the quantity of data presented to the patient, focusing instead on the essential elements. This allows for the inclusion of more substantial content during the counseling session. It is important to note that the perceived importance of certain parameters may fluctuate over time, particularly in the context of CI experience. It is therefore essential that patients are made aware of this phenomenon and are provided with the relevant information. As part of the counseling process, particularly in regard to the selection of the CI System, it is of significant importance to provide patients with a sufficient quantity of information. The use of standardized counseling protocols enables to ensure a defined qualitative standard, but it is still important to address the individual need of the patients in the consultation. Consequently, the CI counseling process is characterized by a high degree of individualization. The clinical routine has demonstrated that CI patients exhibit a considerable degree of variability, both in terms of their underlying etiology and in terms of their technical needs and demands on the CI.

## Data Availability

Data availability is provided by request to the corresponding author.
